# Inhibitory Effect of Trihydroxy Isoflavone on Neuronal Apoptosis in Natural Aging Rats

**DOI:** 10.1155/2022/4688203

**Published:** 2022-08-21

**Authors:** Ke Zhao, Shenghui Li, Jianzhou Chen, Qinghua Jin

**Affiliations:** ^1^Department of Physiology, Baicheng Medical College, Baicheng, 137000 Jilin, China; ^2^Department of Physiology and Pathophysiology, College of Medicine, Yanbian University, Yanji, 133000 Jilin, China

## Abstract

**Objective:**

To explore the impact of genistein (Gen) on the apoptosis of neuronal cells in naturally aged rats and its mechanism.

**Methods:**

Fifty SD male rats were allocated into five groups at random, including youth group (3M group), natural aging group (24M group), and Gen low-, medium-, and high-dose groups. Starting from 18 months of age, Gen 10, 30, and 60 mg-kg^−1^ were administered via gavage to the Gen low-, medium-, and high-dose groups, respectively, while the rats in the natural aging group was given saline by gavage until 24 months of age, and the drug was stopped for 1 d per week for 6 months. The protein expression of target genes was examined using western blotting.

**Results:**

In contrast to the 3M group, the 24M group rats showed disturbed neuronal cell arrangement and massive cell degeneration. After 6 months of Gen intervention, in contrast to the 24M group, the neural cell pathology in the CA3 area of the hippocampus improved and cell apoptotic decreased observably. In contrast to the 3M group, the protein expression of c-Jun amino-terminal kinase (p-JNK), C/EBP homologous protein (CHOP), inflammatory vesicle 3-associated factor (NLRP3), cysteine protease-1 (Caspase-1), and apoptosis-related punctate protein (ASC) and downstream inflammatory factors in the hippocampus was obviously increased in the 24M group. In contrast to the 24M group, the protein expression of p-JNK, CHOP, NLRP3, Caspase-1, and ASC and downstream inflammatory factors in the hippocampus was observably declined in Gen groups.

**Conclusion:**

Gen has a protective effect on hippocampal neurons in aging rat brain tissue via the inhibition of the ERS apoptotic signaling pathway and NLRP3 inflammatory vesicle activation.

## 1. Introduction

Aging is a natural and complex process that is inevitable in the organism; neurodegenerative diseases are its main clinical manifestations [1–3]. With the intensive research on neurodegenerative diseases associated with brain aging, it has been confirmed that oxidative stress, inflammation, and other induced neuroapoptosis are essential in these diseases [4, 5]. Endoplasmic reticulum stress- (ERS-) induced apoptosis is a factor in the development of many neurodegenerative diseases and causes neurological disorders mainly due to oxidative stress and metabolic disorders caused by the accumulation of unfolded proteins [6]. There is evidence that excessive activation of ERS pathway unfolded protein signaling is detected in the brains of AD, PD, and HD patients, inducing apoptosis [7]. Mercado et al. found significantly elevated expression of genes related to the ERS signaling pathway in PD patients [8]. Neuroinflammation-induced apoptosis is involved in aging and neurodegenerative diseases. Increased release of inflammatory factors and other neurotoxic factors has been reported in naturally aging individuals and AD and PD brains [9]. Griffioen et al. found that inhibition of neuroinflammation-related genes significantly prolonged the lifespan of HD mice and that expression of ERS and neuroinflammatory response-related factors was significantly increased in the brains of naturally aging APP/PS1 mice [10].

Genistein (Gen) is an isoflavone rich in soybeans and possesses a variety of biological activities and pharmacological effects, such as antitumor, antifungal, hypolipidemic, and hypoglycemic activities [11]. Numerous studies proved that Gen has a protective effect on nerve cells [12]. Xu et al. found that Gen significantly improved the morphology of vertebral cells in the CA1 region of the hippocampus of mice with cerebral ischemia-reperfusion, declined cell apoptosis, and significantly inhibited apoptosis-related proteins such as TNF-*α* inflammatory factor and p-JNK1/2 in brain tissue, exerting a neuroprotective effect [13]. However, whether Gen can reduce apoptosis in naturally aged rats by inhibiting apoptosis-related proteins of the ERS signaling pathway and inhibiting the inflammatory response activated by NLRP3 inflammatory vesicles remains to be further investigated.

## 2. Materials and Methods

### 2.1. Animals

SPF male SD rats (200 ± 20 g) were acquired from the Laboratory Animal Center of Three Gorges University, Yichang, Hubei, China. The animal production license number is (SCXK)(E) 2011-0012. The feeding environment was 23 ± 3°C, 60 ± 5% relative humidity, 12 h/12 h alternating light and dark, and free feeding and drinking.

### 2.2. Drugs and Reagents

Gen (purity > 95%), Chengdu Phytobiotech Co., Ltd., Lot No. PCS0841; rabbit multiple antibodies JNK (Lot No. #9252), rabbit multiple antibodies p-JNK (Lot No. #9251), rabbit multiple antibodies *β*-actin (Lot No. 4970L), Cell Signaling, USA; Rabbit Multi-Anti-ASC (Lot #: sc-22514-R), Rabbit Multi-Anti-IL-18 (Lot #: sc-7954), Rabbit Multi-Anti-IL-1*β* (Lot #: sc-7884), Sheep Multi-Anti-TNF-*α* (Lot #: sc-1350), Mouse Monoclonal Anti-CHOP (Lot #: sc-56107), Santa Cruz, USA; Rabbit Multi-Anti-NLRP3 (Lot: ab214185), Abcam, USA; Mouse Monoclonal Antibody Caspase-1 (Lot No. NB100-56565), Novus Corporation, USA; and goat anti-rabbit, goat anti-mouse secondary antibody, and donkey anti-goat secondary antibody, Wuhan Corey Co., were used.

### 2.3. Apparatus

PowerPac200 Western Blotting Electrophoresis Instrument, Bio-Rad, USA; BioshineChemiQ4800 Chemiluminescent Gel Imaging Automated Developer, Shanghai OXO Scientific Instruments Co. Ltd.; TP1020 automatic dehydrator, EG1150H paraffin embedding machine, EG1150C ultrathin slicer, Leica, Germany; and BX53 microscope, Olympus, Japan, were used.

### 2.4. Methodology

#### 2.4.1. Animal Grouping and Drug Administration

Ten 3-month-old rats were utilized as the young control group (3M group), and 40 18-month-old rats were allocated into the aging group (24M group), with the Gen low-, medium-, and high-dose groups at random. Starting from 18 months of age, Gen 10, 30, and 60 mg-kg^−1^ were administered by gavage to the Gen low-, medium-, and high-dose groups, respectively, while the natural aging group was given saline by gavage until 24 months of age, and the drug was stopped for 1 d per week for 6 months. The aging group was gavaged with the same amount of saline.

#### 2.4.2. Morphological Changes Observed via HE Staining

The rats were anesthetized with 2.5% pentobarbital sodium through intraperitoneal injection, the blood was flushed by perfusion of saline into the thoracic aorta and then perfused with 4% paraformaldehyde, and the brain was fixed in 4% paraformaldehyde by severing the head. After dehydration, the tissue was paraffin-embedded, 4 *μ*m sections were stained using HE, and the morphology was analyzed microscopically.

#### 2.4.3. Measurement of Apoptosis in Hippocampal Neurons of Aging Rats Using Tunel Method

The cells with brown granules in the nucleus were considered as apoptotic cells, and the results were observed under the microscope.

#### 2.4.4. Detection of ERS- and Neuroinflammation-Induced Apoptosis Using Western Blot

The protein expression of ERS-associated proteins CHOP and p-JNK and neuroinflammation-associated factors was detected. Total proteins were extracted from rat hippocampal tissues, denatured in a water bath at 95°C for 10 minutes, separated through SDS-PAGE gel electrophoresis, and transferred to a membrane, which was incubated using primary antibodies overnight at 4°C. Secondary antibody was put in and incubated at 25°C. Protein bands were developed in ECL and were analyzed by grayscale scanning using ImageJ software.

### 2.5. Statistical Methods

Data analysis was conducted via SPSS 18.0 software. The difference between groups was analyzed using one-way ANOVA. *P* < 0.05 indicated obvious difference between the groups.

## 3. Results

### 3.1. Effect of Gen on the Morphology and Apoptosis of Neuronal Cells in the Hippocampal Region of Naturally Aged Rats

**Figure 1:**
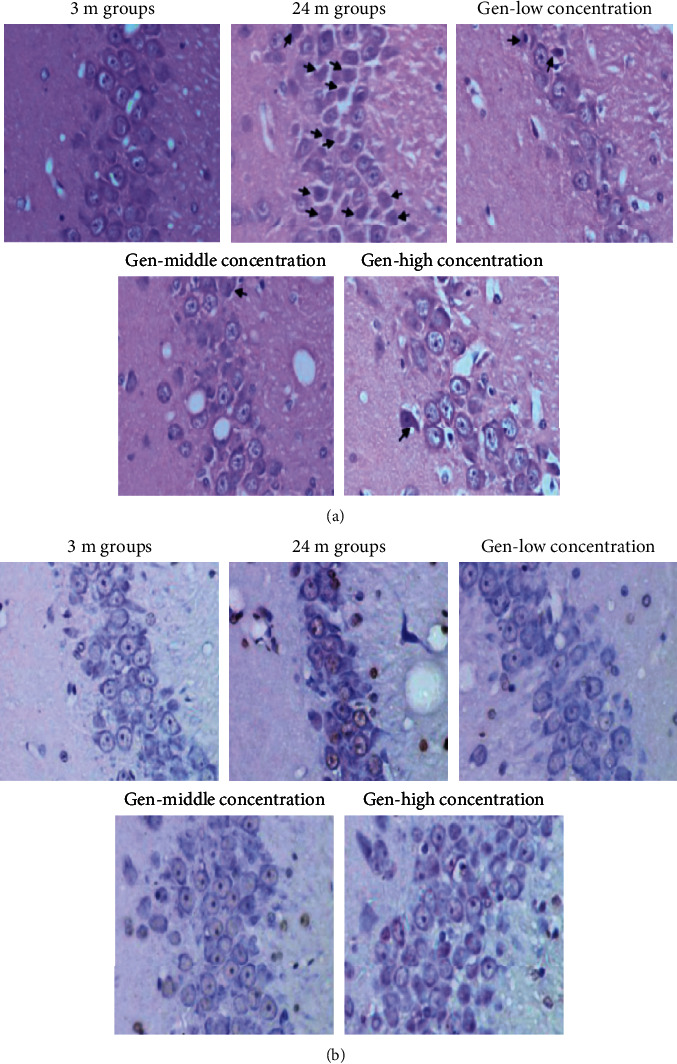
Effect of Gen on the morphology and apoptosis of rat hippocampal neurons (×400). Note: (a) HE staining and (b) Tunel staining. The arrows meant the degenerated cells.

HE (Figure 1(a)) and Tunel (Figure 1(b)) staining was used to observe the pathological changes and apoptosis of neuronal cells in the CA3 region of the hippocampus of rats in each group. The results showed the neuronal cells in the CA3 region of the hippocampus of rats in the 3M group were neatly arranged and tightly packed, with clear nuclei and large, round nuclei; the neuronal cells in the hippocampus of rats in the 24M group were disorganized, with numerous degenerated cells (arrow), and the nuclei of degenerated neuronal cells were solidly and deeply stained in a triangular or irregular shape, and the number of apoptotic cells was high. After 6 months of Gen intervention, in contrast to the 24M group, the neural cell pathology in the CA3 area of the hippocampus improved and the number of apoptotic cells observably declined.

### 3.2. Effect of Gen on ERS-Related Proteins p-JNK and CHOP

**Figure 2:**
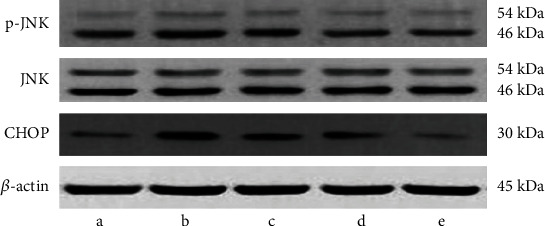
Effect of Gen on p-JNK and CHOP protein expression. Note: (a) 3M group; (b) 24M group; (c) Gen low-dose group; (d) Gen medium-dose group; (e) Gen high-dose group.

The protein expression of p-JNK and CHOP in hippocampal tissues was observed via western blot method. In contrast to those in the 3M group, the protein expression of p-JNK and CHOP was observably raised in the 24M group (*P* < 0.05 and *P* < 0.001, Table 1 and Figure 2). After 6 months of Gen intervention, the protein expression of p-JNK and CHOP was observably downregulated in all Gen dose groups in contrast to the 24M group (*P* < 0.05, *P* < 0.01, and *P* < 0.001; Table 1 and Figure 2).

### 3.3. Effect of Gen on the Protein Expression of Inflammatory Factors

**Figure 3:**
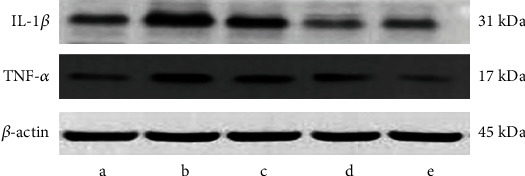
Effect of Gen on the protein expression of inflammatory factors. Note: (a) 3M group; (b) 24M group; (c) Gen low-dose group; (d) Gen medium-dose group; (e) Gen high-dose group.

IL-1*β* and TNF-*α* expression was markedly raised in the hippocampus of the 24M group compared with the 3M group (*P* < 0.01 and *P* < 0.001, Table 2 and Figure 3). All Gen dose groups significantly downregulated IL-1*β* and TNF-*α* in contrast to the 24M group (*P* < 0.05, *P* < 0.01, and *P* < 0.001; Table 2 and Figure 3).

### 3.4. Effect of Gen on the Expression of NLRP3, ASC, Caspase-1, and IL-18

**Figure 4:**
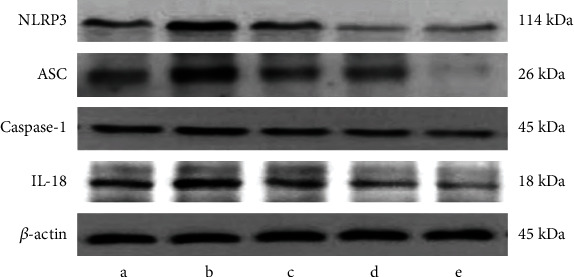
Effect of Gen on the expression of NLRP3 inflammatory vesicle-associated protein. Note: (a) 3M; (b) 24M; (c) Gen low-dose group; (d) Gen medium-dose group; (e) Gen high-dose group.

In contrast to the 3M group, the protein expression of NLRP3, ASC, Caspase-1, and IL-18 in hippocampal tissues was apparently raised in the 24M group (*P* < 0.05 and *P* < 0.01, Table 3 and Figure 4). After 6 months of Gen intervention, the protein expression of NLRP3, ASC, Caspase-1, and IL-18 was observably downregulated in all Gen dose groups in contrast to the 24M group (*P* < 0.05, *P* < 0.01, and *P* < 0.001; Table 3 and Figure 4).

## 4. Discussion

Aging puts the body in a state of chronic stress, and excessive activation of unfolded protein signaling in the ERS pathway triggers apoptosis and neuroinflammation, which are essential in many neurodegenerative diseases [14, 15]. ERS is a cellular response process triggered by various stimuli that disrupt protein folding in the endoplasmic reticulum. However, prolonged and sustained ERS can lead to cell damage and eventually apoptosis. JNK is a downstream apoptotic molecule activated by the IRE1 apoptotic pathway of the three UPR pathways, which mediates the stress response and the apoptotic response induced by inflammatory factors such as TNF-*α* [16]. CHOP is an ERS-specific transcription factor and is one of the most important mediators of the ERS-induced apoptotic pathway [17]. It was found that JNK and CHOP are also involved in regulating apoptosis in neuronal cells. In the literature, it has been reported that neuronal cells in the CA1 region of the hippocampus of aged rats are heavily degenerated and ERS pathway-related proteins such as CHOP and p-JNK are upregulated in the hippocampus and cortex. The present study demonstrated that neuronal cells in the hippocampus of naturally aged rats were heavily degenerated and cell apoptotic was raised, and the expression of p-JNK and CHOP was obviously raised, while Gen significantly improved the morphology of hippocampal tissue, inhibited the expression of apoptotic cytokines p-JNK and CHOP, and reduced the number of apoptotic cells. This suggests that Gen can reduce aging-induced neuronal apoptosis by inhibiting the activation of JNK and CHOP, which are related to the ERS apoptotic signaling pathway.

NLRP3 inflammatory vesicles usually consist of the pattern recognition receptor NLRP3, the junctional protein ASC, and the effector molecule Caspase-1. Upon pathogen invasion, NLRP3 recruits Caspase-1 precursors via the junctional protein ASC and promotes the activation of Caspase-1, which further enhances the inflammatory factor secretion [18]. In turn, the release of inflammatory factors can further promote the release of TNF-*α*, and the activation of excessive inflammatory factors further induces cell death. It has been widely reported in the literature that NLRP3 inflammatory vesicles are extensively involved in the onset and development of aging and neurological diseases. It was found that NLRP3 inflammatory vesicle activation was significantly increased in hippocampal tissue of decompensated rats, and the protein expression of inflammatory factors was also markedly raised, and the apoptosis of neuronal cells was significantly raised. Lee et al. found that toxic *β*-amyloid activates NLRP3 inflammatory vesicles and leads to AD pathology and tissue damage [19], and inhibition of Caspase-1 reduces the activation of RAW264.7. Inhibition of Caspase-1 reduced the activation of BV2 microglia by activated RAW264.7 macrophage inflammatory response and significantly raised the protein expression of NLRP3 inflammatory vesicles and inflammatory factors in the brain of PD model rats. The present study demonstrated the protein expression of NLRP3 inflammatory vesicles and inflammatory factors were observably higher in hippocampal tissues of naturally aged rats in contrast to those of young rats, and Gen significantly downregulated the protein expression of NLRP3 inflammatory vesicles and downstream inflammatory factors. This suggested Gen may play a protective role in inhibiting neuronal apoptosis by suppressing the activation of NLRP3 inflammatory vesicles and reducing the release of inflammatory factors. In conclusion, aging can cause apoptosis in rat hippocampal tissue, and the protective effect of Gen on neuronal cells may be related to the inhibition of the expression of p-JNK and CHOP, the proteins related to the ERS apoptotic signaling pathway, and the inhibition of NLRP3 inflammatory vesicles, thus reducing neuronal apoptosis. There were some limitations in our study. (1) All test indicators were at the end of this experiment, but the aging kinetics has not been confirmed. (2) This study focused on the detection of apoptotic proteins and pathways and lacks the detection of other methods. (3) The limited number of research animals may affect the experimental results. We will consider all the above points in the future.

## Figures and Tables

**Table 1:** Effect of Gen on p-JNK and CHOP protein expression levels ($\bar{x}\pm s$, *n* = 10).

Group	Dose (mg-kg^−1^)	p-JNK	CHOP
3M group	—	0.82 ± 0.06	0.42 ± 0.04
24M group	—	1.05 ± 0.04^∗^	0.74 ± 0.05^∗∗∗^
Gen low-dose group	10	0.81 ± 0.08^#^	0.62 ± 0.04^#^
Gen medium-dose group	30	0.78 ± 0.12^#^	0.55 ± 0.01^##^
Gen high-dose group	60	0.75 ± 0.16^#^	0.34 ± 0.05^###^

Note: in contrast to the 3M group, ^∗^*P* < 0.05 and ^∗∗∗^*P* < 0.001; in contrast to the 24M group, ^#^*P* < 0.05, ^##^*P* < 0.01, and ^###^*P* < 0.001. The following table is the same.

**Table 2:** Effect of Gen on protein expression of IL-1*β* and TNF-*α* ($\bar{x}\pm s$, *n* = 10).

Group	Dose (mg-kg^−1^)	IL-1*β*	TNF-*α*
3M group	—	0.59 ± 0.06	0.47 ± 0.16
24M group	—	0.91 ± 0.17^∗∗^	1.11 ± 0.14^∗∗∗^
Gen low-dose group	10	0.63 ± 0.06^∗∗^	0.85 ± 0.061^#^
Gen medium-dose group	30	0.57 ± 0.08^#^	0.76 ± 0.07^##^
Gen high-dose group	60	0.53 ± 0.04^###^	043 ± 0.17^###^

**Table 3:** Effect of Gen on NLRP3 inflammatory vesicle-associated protein expression ($\bar{x}\pm s$, *n* = 10).

Group	Dose (mg-kg^−1^)	NLRP3	ASC	Caspase-1	IL-18
3M group	—	0.35 ± 0.04	0.83 ± 0.17	1.03 ± 0.07	0.78 ± 0.11
24M group	—	0.57 ± 0.02^∗∗^	1.21 ± 0.03^∗^	1.35 ± 0.20^∗^	1.07 ± 0.13^∗^
Gen low-dose group	10	0.42 ± 0.02^∗^	0.85 ± 0.10^#^	1.05 ± 0.05^#^	0.78 ± 0.09^#^
Gen medium-dose group	30	0.32 ± 0.05^##^	0.88 ± 0.16^##^	0.94 ± 0.02^##^	0.79 ± 0.12^#^
Gen high-dose group	60	0.33 ± 0.10^##^	0.54 ± 0.14^###^	0.96 ± 0.02^##^	0.59 ± 0.07^##^

## Data Availability

Data to support the findings of this study is available on reasonable request from the corresponding author.
